# The immunological interplay between vaccination and the intestinal microbiota

**DOI:** 10.1038/s41541-023-00627-9

**Published:** 2023-02-23

**Authors:** Petra Zimmermann

**Affiliations:** 1 Department for Community Health, Faculty of Science and Medicine, University of Fribourg, Fribourg, Switzerland; 2 Department of Paediatrics, Fribourg Hospital, Fribourg, Switzerland; 3 Department of Paediatrics, The University of Melbourne, Parkville, VIC Australia; 4 Infectious Diseases Research Group, Murdoch Children’s Research Institute, Parkville, VIC Australia

**Keywords:** Medical research, Biomarkers

Vaccination is the most cost-effective life-saving medical intervention^1^. However, substantial variation in individual immune responses to vaccination exists^2^. Lower vaccine immunogenicity, especially to oral vaccines, is often reported in developing countries^3–5^. Many intrinsic and extrinsic factors, such as age, genetics, pre-existing immunity, nutritional status and comorbidities contribute to the variations in vaccine responses^6^.

Growing evidence shows that the composition of the intestinal microbiota, which is known to play an important role in the development and regulation of immune responses^7^, influences responses to vaccination^8–10^. Studies which have investigated this have shown that a higher relative abundance of the phylum Actinobacteria is consistently associated with higher vaccine responses and a higher relative abundance of Bacteroides with lower responses, while the association between the relative abundance of the phyla Firmicutes and Proteobacteria and vaccine responses varies for different genera and species^11–18^. Almost all of these studies investigated the association for oral vaccination^11,12,14–18^, only three studies investigated responses after intramuscular vaccination^13,19,20^.

Only a few studies have investigated whether the administration of vaccines is associated with changes in the composition of the intestinal microbiota^12,20–26^, and whether the changes induced by vaccination, in turn, influence vaccine immunogenicity^20^. These studies have reported inconsistent results. In humans, the administration of oral typhoid or an intranodal and intramuscularly human immunodeficiency virus (HIV)-1 vaccine was not reported to significantly affect the composition of the intestinal microbiota one week to several months after vaccination^12,21,22^. The first two studies used amplicon sequencing, while the third used shotgun metagenomic sequencing. A recent study using metagenomic sequencing, reported that the intramuscular administration of an inactivated and an mRNA SARS-CoV-2 vaccine was associated with lower bacterial diversity, an increase in the relative abundance of *Bacteroides caccae* and a decrease in the relative abundance of *Clostridiales* (*Coprococcus comes, Dorea longicatena* and *Ruminococcus obeum*) in the intestinal microbiota one month after vaccination^20^. Animal studies have also shown that the administration of vaccines can lead to changes in the intestinal microbiota: in mice, an increase in the relative abundance of *Bacteroides* has been reported after intramuscular vaccination with a *Mycobacterium tuberculosis* vaccine^25^ and compared with mice vaccinated with a placebo, mice vaccinated intramuscularly with an HIV T-cell immunogen had a higher relative abundance of *Clostridiales (Eubacterium xylanophilum, Roseburia* and *Ruminococcus*)^26^. In rhesus macaques, the intradermal administration of a combined HIV-1 DNA/protein vaccine has been shown to lead to an increase in the Firmicutes/Bacteroidetes ratio and a decreased relative abundance of *Prevotella, Alloprevotella, Bacteroides, Acetobacteroides, Falsiporphyromonas* and *Anaerocella*. Interestingly, the abundance of *Prevotella* negatively correlated with rectal HIV-1 specific immunoglobulin G levels^24^. In piglets, the administration of an oral *Lawsonia interacellularis* vaccine led to an increased relative abundance of *Streptococcus* and *Ruminococcus*, and a decreased relative abundance of *Clostridium*^23^.

**Fig. 1:**
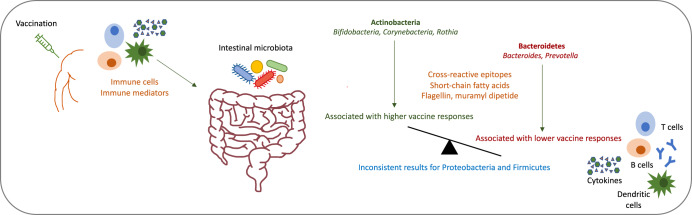
Proposed mechanisms by which vaccination might influence the composition of the intestinal microbiota and by which the intestinal microbiota might influence vaccine responses.

The proposed mechanisms behind the influence of the composition of the intestinal microbiota on vaccine immunogenicity include cross-reactive epitopes between microbes and vaccine antigens, modulation of B cell responses through microbial metabolites such as short-chain fatty acids (SCFAs), and the provision of natural adjuvants through certain microbes (Fig. 1)^10^. The latter includes the stimulation of pattern recognition receptors through bacterial components, such as the stimulation of toll-like receptors (TLR)-5 through flagellin^27^ or nucleotide-binding oligomerization domain (NOD)-2 receptors through muramyl dipeptide which is part of peptidoglycan^28^. A recent study shows that “silent” flagellins, which are weak TLR-5 agonists are abundant in Lachnospiraceae in the intestinal microbiota and overrepresented in non-industrialised populations^29^. The microbiota has also been shown to regulate type I interferon production by plasmacytoid dendritic cells, which stimulates conventional dendritic cells to more efficiently prime antigen-specific T cell responses^30^. Moreover, in mice, SCFAs (acetate, propionate and butyrate) produced by microbes of the intestinal microbiota through fermentation of dietary fibres have been shown to enhance B cell metabolism and regulate gene expression to promote B cell differentiation into antibody-producing cells. Mice with low dietary fibre intake or microbial insufficiency were defective in producing pathogen-specific antibody responses, which could be restored by the intake of SCFAs^31^. As a further example, the abundance of the genus *Roseburia*, a butyrate-producer, has been positively correlated with circulating interleukin (IL)-27 levels and T cell responses to an HIV T cell vaccine^26^.

Little is known about the mechanisms by which vaccination might induce changes in the intestinal microbiota. Immune cells and inflammation mediators induced by vaccination, likely migrate with the blood flow from the vaccination side to the intestine, where they interact with microbes (Fig. 1). As an example, it has been hypothesised that vaccination with an HIV T cell vaccine leads to T helper 1 cell stimulation and increased levels of inflammation mediators, which might lead to an increase in the abundance of *Roseburia* in the intestinal microbiota^26^. If T cell responses play an important role in inducing changes in the microbiota after vaccination, a further potentially important vaccine which could change the composition of the intestinal microbiota, is Bacillus Calmette-Guérin (BCG) vaccine. BCG is one of the most widely used vaccines globally and it is known to have many non-specific effects.

The limitations of the studies investigating the association between vaccination and changes in the composition of the intestinal microbiota and vice versa include small cohort size; the lack of longitudinal sampling which would allow for better assessment of temporal variations; a large heterogeneity in study design, including different vaccine and vaccine schedules, and timing of collection between vaccination and stool and blood samples. Furthermore, different analysis techniques have been used, and results have been reported on different taxonomic levels. Many studies have used amplicon sequencing which might not be sensitive enough to detect small differences in microbial composition. Moreover, there is a lack of investigations on the mechanisms by which vaccination might induce changes in the intestinal microbiota. This would require not only to investigate the composition of the intestinal microbiota, but also translational approaches, including in vitro and in vivo experiments.

Understanding how vaccines might change the composition of the intestinal microbiota and how these changes could influence vaccine responses, is crucial to optimise vaccine responses, especially in settings or individuals in which/whom lower immunogenicity is expected, such as in developing countries or after antibiotic exposure^32^. Bacterial species identified to positively influence vaccine responses could be administered as probiotics before vaccination in individuals who are at risk for low vaccine immunogenicity^33^. Further interventions to possibly optimise the microbiota composition to improve vaccine efficacy include the administration of synbiotics (a mixture of pro- and prebiotics), faecal transplants and small molecules that interact with specific bacterial processes. Importantly, as the composition of the intestinal microbiota has been associated with the risk of many immune- and non-immune-mediated diseases, vaccination could also be used to optimise the composition of the microbiota, for example in infants who are at risk for allergic diseases and asthma^34^.
